# Coping with Reactive Oxygen Species to Ensure Genome Stability in *Escherichia coli*

**DOI:** 10.3390/genes9110565

**Published:** 2018-11-21

**Authors:** Belén Mendoza-Chamizo, Anders Løbner-Olesen, Godefroid Charbon

**Affiliations:** Department of Biology, University of Copenhagen, Ole Maaløes vej 5, 2200 Copenhagen N, Denmark; b.m.chamizo@bio.ku.dk (B.M.-C.); lobner@bio.ku.dk (A.L.-O.)

**Keywords:** *Escherichia coli*, DNA replication, genome stability, oxidative damage, ROS

## Abstract

The facultative aerobic bacterium *Escherichia coli* adjusts its cell cycle to environmental conditions. Because of its lifestyle, the bacterium has to balance the use of oxygen with the potential lethal effects of its poisonous derivatives. Oxidative damages perpetrated by molecules such as hydrogen peroxide and superoxide anions directly incapacitate metabolic activities relying on enzymes co-factored with iron and flavins. Consequently, growth is inhibited when the bacterium faces substantial reactive oxygen insults coming from environmental or cellular sources. Although hydrogen peroxide and superoxide anions do not oxidize DNA directly, these molecules feed directly or indirectly the generation of the highly reactive hydroxyl radical that damages the bacterial chromosome. Oxidized bases are normally excised and the single strand gap repaired by the base excision repair pathway (BER). This process is especially problematic in *E. coli* because replication forks do not sense the presence of damages or a stalled fork ahead of them. As consequence, single-strand breaks are turned into double-strand breaks (DSB) through replication. Since *E. coli* tolerates the presence of DSBs poorly, BER can become toxic during oxidative stress. Here we review the repair strategies that *E. coli* adopts to preserve genome integrity during oxidative stress and their relation to cell cycle control of DNA replication.

## 1. Introduction

**Figure 1:**
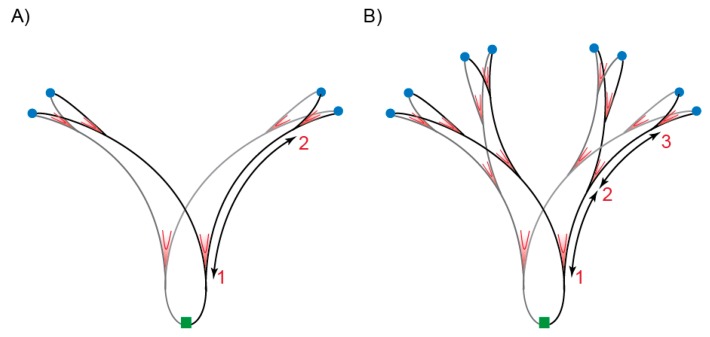
Distribution of replication forks during normal replication and hyper-replication. Schematic representation of a chromosome in the process of replication in cells growing with a doubling time of ~30 min; (**A**) normal replication and (**B**) hyper-replication. Origin of replication (*oriC*) regions are marked in blue and the terminus region in green. Ongoing replication forks are indicated in red (V marks). Numbers indicate the number of replication rounds. The distance between replication forks, represented by arrows, is shorter in hyper-replicating cells.

Depending on nutrient conditions, *Escherichia coli* can grow with a generation time of several hours or less than 20 min. Classically, the cell cycle of slow-growing cells (more than one hour doubling time) is represented in three phases: after birth the pre-replication period (B) starts during which cells increase their size without duplicating their DNA, then comes the replication period (C) during which the chromosome is duplicated and finally the division period (D) corresponding to the time between the termination of DNA synthesis and cell division [1]. In slow growing cells, C and D periods vary with growth rate between ~40 to ~100 min and ~20 to ~60 min, respectively. When bacteria are growing fast, C and D periods are almost constant and last about 40 min and 20 min, respectively [2]. During fast growth, there is no B period and because the cellular doubling time is shorter than the time required to duplicate the chromosome and divide (C plus D), cells are born with chromosome in the process of being replicated. In this case, DNA replication is initiated in the mother, grandmother or great-grandmother cell, depending on the growth rate. DNA replication commences at a unique origin of replication (*oriC*) and proceeds bidirectionally on the circular chromosome until replication forks meet at the terminus region situated opposite of *oriC* on the chromosome. Regardless of how fast cells grow, DNA replication is initiated at a constant mass per origin (cell mass/*oriC*) called the initiation mass [3]. This phenomenon is primarily explained by the necessity to accumulate a fixed amount of active ATP-bound DnaA initiator protein in order to start replication [4]. At initiation, DnaA–ATP forms one or two filaments on *oriC* [5], which in turn results in assembly of two replication machineries to start DNA replication. The mechanisms controlling DnaA activity and/or its access to *oriC* directly or indirectly are quite complex, but our understanding is that the accumulation of the initiator in its active form (bound to ATP) plays an essential role while the inactive form of the initiator (bound to ADP) would have an inhibitory role, although this is debated (for review [6,7,8]). Interestingly, because of the way the cell cycle is built, DnaA and/or replisome activities determine when cells divide: if initiation of DNA replication is delayed or the C period is prolonged, the cells divide later (the cells become longer). Another corollary of *E. coli* cell cycle architecture is that because initiation happens once per cell cycle and at all origins present in the cell, each initiation events will be separated by one generation time. This is especially pertinent when considering fast growing cells with doubling times shorter than the C period that can contain up to six forks replicating a chromosome (Figure 1A). Each of these is initiated at *oriC* one doubling time before the following one. Cells are born with even more replication forks when the C period is prolonged, such as when DNA replication is challenged by lowering deoxynucleotide triphosphate (dNTP) abundance.

The bacterium’s ability to grow fast comes at the price of sensitizing the chromosome to DNA damage. When DNA damage is sensed, cell division is delayed by the SOS response [9]. However, unlike the situation in eukaryotic cells, there is no known check point blocking initiation. In other words, DnaA keeps initiating new rounds of DNA replication, despite the fact that the chromosome is plagued by strand breaks [10,11,12,13,14,15]. This leaves *E. coli* with the daunting task of repairing the chromosome in a relative short time, else replication forks collide and generate strand breaks and/or single-strand breaks that are turned into double-strand breaks (DSB) during DNA synthesis. These DSBs are poorly tolerated by *E. coli* that is effectively killed by the accumulation of only few DSBs in the chromosome [16,17].

*Escherichia coli* faces many conditions in which DNA can be damaged and, like all aerobically living organisms, the bacterium is subject to the potentially lethal oxidation of its DNA (see review [18]). Molecular oxygen does not readily react with the cell components, but its derivatives can become toxic for the cells. Several common oxidants derived from molecular oxygen are found in the environment or even produced by the cellular metabolism. Classically, three reactive oxygen species (ROS) are considered, i.e., superoxide anions (O_2_^−^), hydrogen peroxide (H_2_O_2_) and hydroxyl radicals (HO^•^), but other reactive and abundant molecules exist.

H_2_O_2_ is not a radical species and is chemically less reactive than others, but it has the ability to diffuse through biological membranes and reach the cytoplasm very easily [19,20]. Although on its own H_2_O_2_ does not oxidize DNA, it reacts with the intracellular pool of iron to generate hydroxyl radical (HO^•^) via the Fenton reaction.

(Fe^2+^ + H_2_O_2_ → Fe^3+^ + HO^•^) (1)

HO^•^ is an extremely potent oxidant that instantly reacts with organic molecules located in its vicinity. When H_2_O_2_ reacts with iron bound to DNA, HO^•^ creates DNA damage [21].

Superoxide anions cannot diffuse through membranes because of their charge. Inside the cells, O_2_^−^ is mainly generated by auto-oxidation of flavin-containing enzymes. Like H_2_O_2_, O_2_^−^ does not oxidize DNA directly, but because of its dismutation into H_2_O_2_ and because O_2_^−^ can oxidize iron-containing enzymes and in the process release H_2_O_2_ and iron, O_2_^−^ indirectly feeds the Fenton reaction. Here we will discuss how these ROS affect the stability of *E. coli* genome and how the bacterium prevents or evades toxic DNA lesions.

## 2. Sources of Reactive Oxygen Species

*Escherichia coli* frequently encounters ROS in its environment, typically in the form of H_2_O_2_ generated as a byproduct of metabolic activities of other gut microorganisms such as lactic acid bacteria [22,23], but also when macrophages attempt to eradicate bacteria [24]. Plants and microorganisms also produce antibiotic molecules such as plumbagin that poison *E. coli* by hijacking its metabolism to produce O_2_^−^ in a futile cycle (so-called redox cycling molecules) [25,26,27].

**Figure 2:**
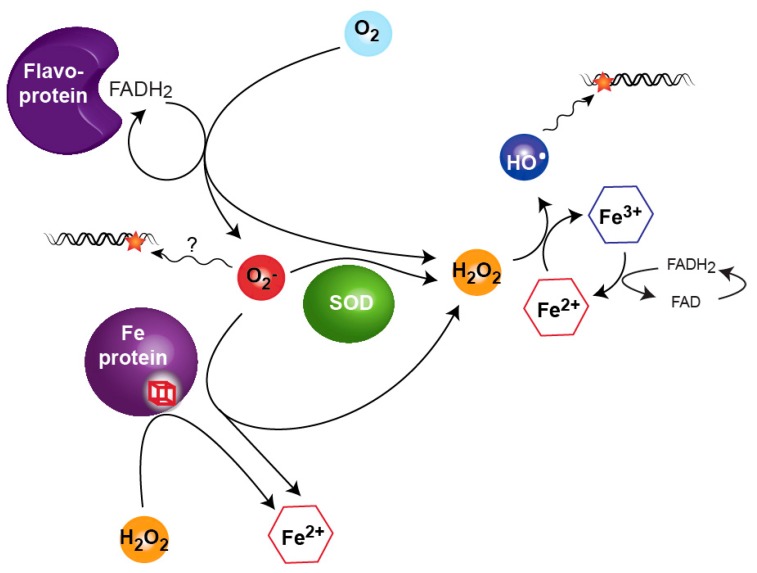
The ability of hydrogen peroxide (H_2_O_2_) and superoxide anion (O_2_^−^) to damage DNA is mostly explained by feeding directly or indirectly the Fenton reaction. The presence of O_2_^−^ increases the level of free Fe^2+^ in the cells by attacking Fe-containing proteins. O_2_^−^ also contributes to H_2_O_2_ release directly when processed by superoxide dismutase (SOD) and indirectly by oxidation of Fe in proteins. H_2_O_2_, through the Fenton reaction, generates free Fe^3+^ and hydroxyl radical (HO^•^) that subsequently damages DNA.

However, ROS are also continuously produced endogenously as a toxic byproduct of the cellular metabolism. Although historically depicted as the main culprit in other organisms, the respiratory chain does only account for a minor part of the self-generated ROS in *E. coli*. This is inferred from the fact that respiration-deficient strains do not produce less ROS [28]. The current model is that menaquinone, flavoproteins and enzymes with cytoplasmic exposed iron sulfur cluster are the main culprits (Figure 2).

## 3. Sensing Reactive Oxygen Species

In normal aerobic conditions, superoxide dismutases are abundant in cells. In other words, the bacterium faces a significant threat by growing in atmospheric concentration of oxygen. This is obvious when looking at catalase/peroxidases or superoxidases deficient mutants that are absolutely dependent on recombination DNA repair functions during aerobic growth, suggesting that low concentrations of O_2_^−^ or H_2_O_2_ are capable of inflicting DNA damage [29,30]. *E. coli* possesses two systems to respond to stressing levels of ROS, OxyR (Oxidative stress Regulator) being dedicated to face high levels of H_2_O_2_ and SoxR/S (SuperOXide Response) reacting to high levels of redox cycling molecules that generate O_2_^−^ (see review [31]). Surprisingly, there is little overlap between OxyR and SoxR/S regulons. This comes as a surprise because O_2_^−^ generates H_2_O_2_ (Figure 2), therefore O_2_^−^ should logically induce both SoxR/S and OxyR. This is not the case in conditions where *E. coli* is hyper-oxygenated or treated with small doses of paraquat; in these conditions only SoxR/S is turned on, while OxyR is not [32,33]. There is however a common theme to the response triggered by OxyR and SoxR/S: (1) the induction of detoxifying enzymes catalase and peroxidases in the case OxyR and of a superoxide dismutase in the case of SoxR/S; (2) the reduction of the pool of available Fe^2+^, among other things by inducing the expression of the transcription regulator Fur (Ferric Uptake Regulation); (3) the expression of oxidation-resistant isozymes, with a net result of restoring essential metabolic activities and/or preventing the leaching of iron from ROS-damaged Fe-containing enzymes.

But what is astonishing is the quasi absence of response to DNA damage. OxyR induces the expression of Dps (DNA binding-protein from starved cells) and SoxR/S induces the expression of the AP endonuclease Nfo (see below). Since the DNA damage inflicted by H_2_O_2_ and O_2_^−^ depends on the formation of HO^•^, with therefore identical consequences in principle, it is intriguing that the strategies used to protect and repair the DNA are different.

## 4. A Metal-Poisoning Disease 

Like all organisms, *E. coli* has to adapt its redox balance to oxygen and iron availability. Oxidative stresses are primarily viewed as a breakdown of iron homeostasis. Removing iron or limiting its reduction in the cell prevents the Fenton reaction to occur and limits DNA damage. Therefore, in wild type cells the control of iron trafficking, its reduction and storage becomes essential. Mutations or conditions that increase the level of free iron in the cells are detrimental while lowering its level is protective [30,34,35]. Fur is the master regulator of iron acquisition that directly senses the cytoplasmic level of iron and affects the expression of iron transporters and iron storage proteins. During aerobic growth, the role of Fur is normally to prevent the overuse of iron. Inactivating Fur function in superoxide dismutase or catalase/peroxidases deficient cells severely aggravates the oxidative stress [30,36]. Consistent with this idea, a Fur mutant is dependent on active DNA repair/recombination unless they are grown anaerobically [30,34,35]. Furthermore, the mutant´s phenotype can be reversed by overproducing iron storage proteins, by inactivating iron transport proteins or by addition of an iron chelator or antioxidant to the growth medium [30,34,35]. The only protein directly involved in genome stability induced by OxyR in response to H_2_O_2_ is the iron-chelating DNA-binding protein Dps [37,38]. Dps is a non-specific DNA-binding protein that has ferroxidase catalytic activity [39]. During H_2_O_2_ stress, Dps protects the chromosome by degrading H_2_O_2_ and chelating iron at the same time. Because it binds and condenses the chromosome, Dps, like other nucleoid-associated proteins, was expected to have a profound impact on DNA and RNA synthesis. However, Dps has only a minor effect on initiation of DNA replication and possibly also on the rate of DNA synthesis [40]. A recent report indicates that Dps has evolved to form very dynamic complexes in order to permit RNA polymerase access to the DNA [41]. Furthermore, Dps may form a non-lipid organelle, the like of nucleosomes, that could physically separate *E. coli* DNA from its cytoplasm. This feature is quite intriguing and could potentially expand the role of Dps.

**Figure 3:**
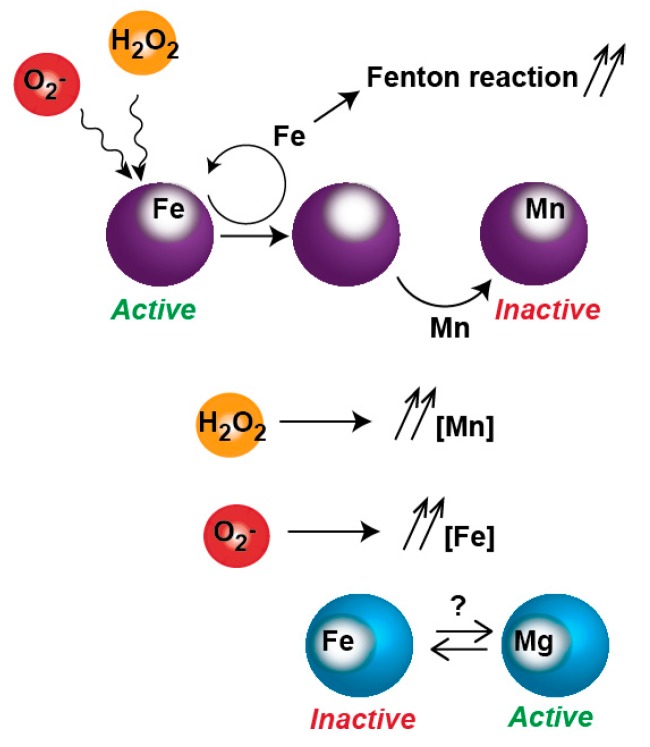
Inactivation of metalloproteins by oxidative stress. Reactive oxygen species (ROS) promotes Fe dissociation from Fe-protein. The apo-protein is re-metallated with Fe or other metals such as Mn. The mis-metallated protein becomes resistant to ROS but is inactive. In O_2_^−^ presence, free Fe is increased, possibly poisoning non-Fe enzymes.

Oxidative stress also induces metal poisoning. ROS can inactivate metalloproteins such as Fur by promoting the dissociation of solvent-exposed iron atoms (Figure 3). These apo-proteins eventually reincorporate iron but also other divalent metals such as Mn^2+^ or Zn^2+^; once ‘mis-metallated’ these enzymes become resistant to O_2_^−^ and H_2_O_2_ oxidation but more importantly they lose their activity [42,43,44]. Part of the response to ROS is directed to reinstate metabolic activities normally performed by ROS-sensitive enzymes, by expressing isozymes that are resistant to oxidation, some of which uses Mn^2+^. To allow for this metallation to occur, *E. coli* specifically induces the expression of a Mn^2+^ transporter. However, as seen in the case of ribonucleotide reductase (RNR) isozymes synthetizing dNTPs [44], this increase in cytoplasmic Mn^2+^ can poison Fe^2+^ enzymes. In other words, the use of oxidant-resistant isozymes could come at the cost of poisoning a set of Fe^2+^ enzymes normally active in absence of oxidative stress. To make the matter even more complicated, free Fe^2+^ becomes poisoning as its cytoplasmic concentration increases during O_2_^−^ stress and feeds the Fenton reaction [45].

## 5. Responding to Oxygen

The metallation of enzymes is linked to the redox status of the cells. This is exemplified by the trio of RNR isozymes used by *E. coli*. Type Ia and Ib RNR are dependent on oxygen to form a radical essential for the enzymatic activity while the activity of type III RNR is destroyed by oxygen. Consequently, the expression of the enzymes is controlled to match the conditions in which the cells grow, Fe^2+^-RNRIa being expressed in normal aerobic condition, Mn^2+^-RNRIb during oxidative stress or when Fe^2+^ is limiting and Fe/S-RNRIII in anaerobic condition [44]. This theme is also seen in proteins of the respiration chain or tricarboxylic acid cycle, allowing for function during oxidative stress. Indirectly, the respiratory chain function becomes important during oxidative stress, as it is expected to limit the time electrons are sitting in ROS-generating proteins or molecules [28]. But *E. coli* can also rely on a broad choice of terminal oxidases to adapt its respiration and/or directly use H_2_O_2_ as a terminal electron acceptor and therefore deplete ROS while also making energy [46,47]. Therefore sensing the redox status of the cells becomes a first line of adaptation to oxidation, especially considering the fact that SoxR/S and OxyR do not sense differences such as the one between aerobic and anaerobic growth. This is in part achieved by ArcB/A system (Anoxic redox control) sensing the redox level of quinones and by FNR (Fumarate Nitrate Reduction) sensing the level of O_2_. Together with Fur, these systems adapt the expression of oxidant-sensitive/resistant isozymes, the flux of electrons in the respiration chain and metal homeostasis [48,49,50], all three systems affecting the expression of the Mn^2+^-superoxide dismutase and HPI catalase that are induced by OxyR and/or SoxR during oxidative stress.

## 6. Repair of DNA Lesions by Base Excision Repair Pathway

Hydroxyradicals can oxidize the ribose or the base moieties of a DNA molecule, resulting in a wide variety of lesions [21]. Because of the low redox potential of guanine, its oxidation into 8-oxo-7,8-dihydroguanine (8-oxoG) and 2-6-diamino-4-hydroxy-5-formamidopyrimidine (FapyG) is assumed to be the most common lesion produced by HO^•^ [51]. 8-oxoG is primarily mutagenic because it can form base pair with adenine leading to G:C -> T:A transversions. 8-oxoG is not a major obstacle for DNA replication in *E. coli* but recent reports indicate that its presence at 3’ ends produces abortive ligation with potential consequences during DNA repair or gap-filling in lagging strand synthesis [52,53]. Oxidative damages such as 8-oxoG or FapyG are normally corrected by base excision repair (BER). The removal of 8-oxoG is mainly carried out by the formamidopyrimidine DNA glycosylase MutM that excises 8-oxoG from the DNA and leaves a single-strand gap. The 3’ phosphate end formed by MutM cannot serve as primer for gap-filling by PolI unless processed by AP endonucleases (see below). The formation of these gaps is the main toxic event derived from the oxidation of guanine. These single-strand gaps can persist in the DNA, and if not repaired in time, become mutagenic or generate DSBs during DNA synthesis [54]. In *E. coli* there are three bifunctional glycosylases associated with oxidative damages: MutM, Nei and Nth. Genetic evidence indicates a certain level of redundancy between the three [55,56] but MutM is mostly specialized in the removal of 8-oxoG and FapyG, while Nei and Nth [57] are primarily associated with the removal of oxidized pyrimidines and FapyA. MutM works in conjunction with MutY and MutT (GO system; see review [58]). While MutY removes adenine mispaired with 8-oxoG, MutT sanitizes 8-oxodGTP nucleotides. The respective functions of the trio can be reflected in the type of mutation acquired when their functions are impaired. The accumulation of transversion mutations G:C -> T:A and A:T -> C:G are characteristic for strains defective in MutY/MutM and MutT respectively [59,60,61]. When cells are exposed to H_2_O_2_, an increase in several types of mutations is observed, G:C -> T:A transversion being preponderant [62,63]. Notably, A:T -> C:G are not increased in these conditions. This indicates that 8-oxoG lesions are inflicted directly in the chromosome rather than by incorporation of 8-oxodGTP nucleotides. As mentioned earlier, single nucleotide gaps are generated by the actions of glycosylases (MutM, Nei, Nth). Depending on the glycosylase activity, after incision of the damaged base, 3’ and or 5′ ends generated are not substrate for DNA PolI/ligase repair. The 3′ phosphate ends generated by MutM and Nei or the 3’phosphate unsaturated aldehyde generated by Nth are restored to 3’ hydroxyl ends by the actions of two AP endonucleases XthA and Nfo, the first one carrying the majority of the activity during normal conditions [64]. Nfo is the only DNA repair enzyme induced by SoxR/S when cells are stressed with high levels of O_2_^−^ [33,65]. Consequently, cells deficient in Nfo are more sensitive in these conditions [66]. Likewise, mutations affecting gap-filling by PolI and ligase render the cells hypersensitive to ROS [67,68,69].

## 7. Toxic Repair

There is substantial evidence that because of its lack of DNA damage check point mechanisms, *E. coli* adopts a strategy that preserves genome stability by tolerating damages. In fact, oxidative stresses do not induce a generic DNA damage response or even the GO system (MutM/Y/T) while other types of stress do [54]. This DNA damage tolerance is essential because gapped DNA generated by repair of ROS-inflicted lesions are in all appearance not easily dealt with. Counterintuitively, cells fare better during genotoxic stress without glycosylases that remove damaged bases and in the process generate DNA gaps [70,71,72,73,74,75,76]. This is exemplified by strains deficient in bifunctional glycosylases (MutM [77] or Nth [78]) becoming more resistant to H_2_O_2_. This is because single-strand gaps are turned into DSBs if replication forks meet them [79]. Therefore, when AP endonuclease (Xth/Nfo) action or gap-filling function are affected, cells become hypersensitive to ROS. In other words, we suggest that the cells have to balance the rate of DNA replication with the rate of BER. Because the spacing between replication forks in *E. coli* is determined by the speed of growth and the initiation frequency, gap site toxicity can be alleviated by growing in poor growth media and/or reducing initiation frequency, for example by limiting DnaA activity or simply inactivating BER [72,80,81,82]. Our model is that even cells growing in normal aerobic conditions keep a minimal time interval between replication forks and that this interval accommodates the repair of oxidative damage. As such, the relative rates of DNA initiation and replication seem to be evolutionarily selected to be in a quite delicate balance, wherein even small perturbations are sufficient to cause problems. This explains the nature of suppressor mutations that restore aerobic growth in cell cycle mutants with an increased number of replication forks (hyper-replicating) [83]. These mutations affect iron/sulfur cluster synthesis, Flavin/Fe reduction, ArcA and/or respiration [82,84]. In the hyper-replicating cells, the gaps generated by MutM are toxic [10] and aerobic growth can be restored in the absence of MutM [10] or when PolI is overproduced [85]. Recombination repair functions are essential under these conditions [86] and the DNA damage seen in hyper-replicating cells is avoided in absence of oxygen. Recently, in an effort to find new antibiotics targeting DNA replication by screening for molecules that restore hyper-replicating cells growth, a well-known iron-chelating molecule, Deferoxamine, was isolated [87]. Indeed, Deferoxamine has been used for decades to prevent DNA damage during O_2_^−^ or H_2_O_2_ stress [88,89,90,91]. We suggest that the strategy devised by *E. coli* to ensure viability during oxidative stress may primarily be one of damage avoidance and in severe cases damage tolerance. Damage tolerance is also seen in the SOS response [9,92] where error-prone translesion polymerases are induced during the later stages, and in the response to a dNTP pool imbalance during genotoxic stress which triggers error-prone DNA replication by PolIII [93,94]. The cells rationale behind damage tolerance seems to be that it is better to risk a certain degree of mutagenesis than face death by failing to repair on time.

## 8. Future Prospect

The sensing of oxidative stress by OxyR and SoxR/S triggers a DNA damage repair/protection response that is both very limited and specific to the ROS. Although H_2_O_2_ and O_2_^−^ are proposed to damage DNA by formation of HO^•^, *E. coli* appears to consider the two ROS as distinct DNA damaging agents. To account for O_2_^−^ specific toxicity towards DNA, a model in which DNA repair proteins are inactivated has been proposed several decades ago [95]. Although this model has since been challenged [96], the recent discoveries that metallo-enzymes can be poisoned during oxidative stress and that AP endonucleases can be affected in such a way could indicate that these activities are limiting or toxic. Indeed, the *in vitro* activity of the housekeeping AP endonuclease in *E. coli* and mammalian cells is inhibited by divalent ions such as Fe^2+^ while the AP endonuclease Nfo, induced by SoxR/S, is unaffected [97]. This situation could be mimicked *in vivo* when free iron concentration is increased during O_2_^−^ stress. Whether the housekeeping AP endonuclease is then poisoned should be reassessed. In this model, Nfo would palliate a deficiency in AP endonuclease activity instead of merely increasing it.

Little is known about the cell cycle parameters of cells facing constant ROS insults or how many cell cycle mutants present oxygen-dependent phenotypes. The cell cycle of *oxyR*, *SoxS*, peroxidase-catalase or superoxide dismutase deficient cells should be investigated and cell cycle mutants reexamined in absence of an oxidant. Finally, several reports now indicate that common lab media generate H_2_O_2_ molecules in quantities sufficiently high to prevent the aerobic growth of peroxide-sensitive strains [98,99,100,101]. Because these media are used to obtain fast growth, it is a possibility that the aerobic sensitivity of certain cell cycle mutants reflects the presence of H_2_O_2_ rather than the speed of growth.
